# Incidental Detection of a Rare Pediatric High-Grade Fibrosarcoma in a Post-traumatic Setting: The Conundrum of Intra-Abdominal Hematoma versus Neoplasia

**DOI:** 10.1155/2023/3178778

**Published:** 2023-12-05

**Authors:** Kamand Khalaj, Nikoo Fattahi, Allen C. Omo-Ogboi, Jaiyeola O. Thomas-Ogunniyi, Olanrewaju A. Ogunleye, Ashish Khanal, Larry A. Kramer

**Affiliations:** ^1^Department of Diagnostic and Interventional Imaging, University of Texas Health Science Center, Houston, USA; ^2^Department of Pathology and Laboratory Medicine, University of Texas Health Science Center, Houston, USA

## Abstract

Infantile fibrosarcoma (IF) is a rare malignant fibroblastic tumor that affects infants and young children, occurring most commonly in the extremities. Here, we present a 14-year-old patient with an abdominal mass incidentally detected after a blunt injury to the abdomen. The initial trauma protocol CT revealed a high attenuation mesenteric lesion in the left central abdomen suggestive of mesenteric hematoma. However, the possibility of a solid neoplastic mass lesion could not be excluded. Further evaluation with dynamic contrast-enhanced serial MRI showed a progressive enhancing mass and excluded a hyperacute hematoma with active bleeding. The mass was resected, and histopathological examination and molecular analysis of tumor cells were consistent with a high-grade fibrosarcoma with KMT2D : BCOR fusion.

## 1. Introduction

MRI is a highly sensitive and specific imaging modality and widely considered the modality of choice for evaluating soft tissue tumors [1]. Despite this advantage, MRI of neoplastic tumors may occasionally mimic the imaging features of non-neoplastic masses or vice versa. For instance, soft tissue hematoma, a common post-traumatic condition, could be misdiagnosed as a neoplasm [2, 3]. Conversely, soft tissue tumors may be misidentified as hematomas because of their similar imaging features [4]. Because of this pitfall, careful analysis of dynamic contrast-enhanced MRI and serial MRI scans may be required to avoid an interpretive error. This case report explores the imaging conundrum of distinguishing an incidental malignant neoplasm from a hematoma.

## 2. Case Report

A 14-year-old patient with no significant past medical history presented to the emergency room after an all-terrain vehicle (ATV) accident. The patient had a handlebar injury to the abdomen. The physical examination revealed tenderness in the abdomen.

**Figure 1:**
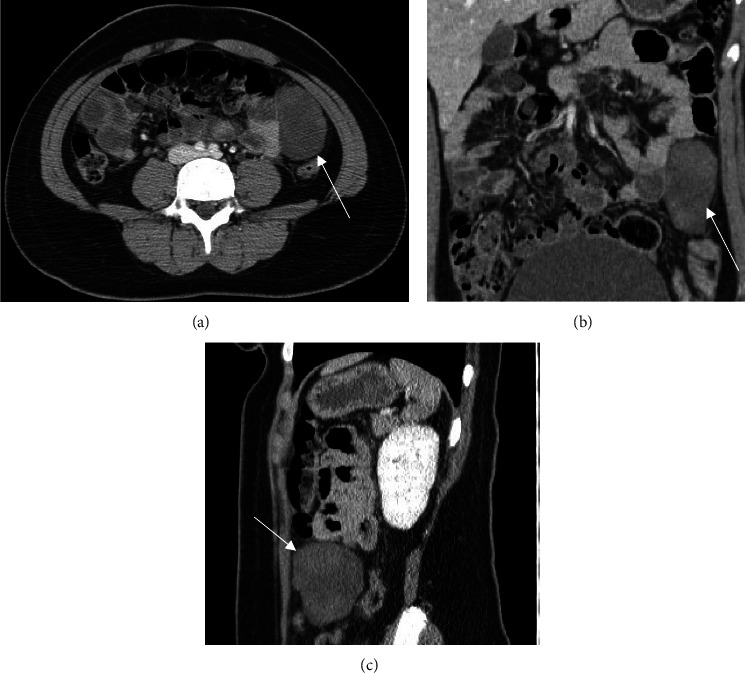
Axial (a), coronal (b), and sagittal (c) view of a contrast-enhanced CT of abdomen which showed a 9 × 5.5 × 3.9 cm smoothly marginated heterogenous hyperdense lesion in the left mid abdomen separate from the adjacent bowel loops (arrows).

Lab investigation showed anemia (Hb = 11.3). However, all other hematological and biochemical analyses were normal and stable. An abdominal CT scan with IV contrast (Figure 1) was performed, which revealed a well-defined 9 × 5.5 × 3.9 cm heterogenous mass with a mean attenuation of 50 HU. Considering the recent history of trauma, the mass was thought to represent a hematoma. However, in the absence of comparative non-contrast images, an underlying malignancy could not be entirely excluded. The patient underwent a dynamic contrast abdominal MRI for further investigation.

**Figure 2:**
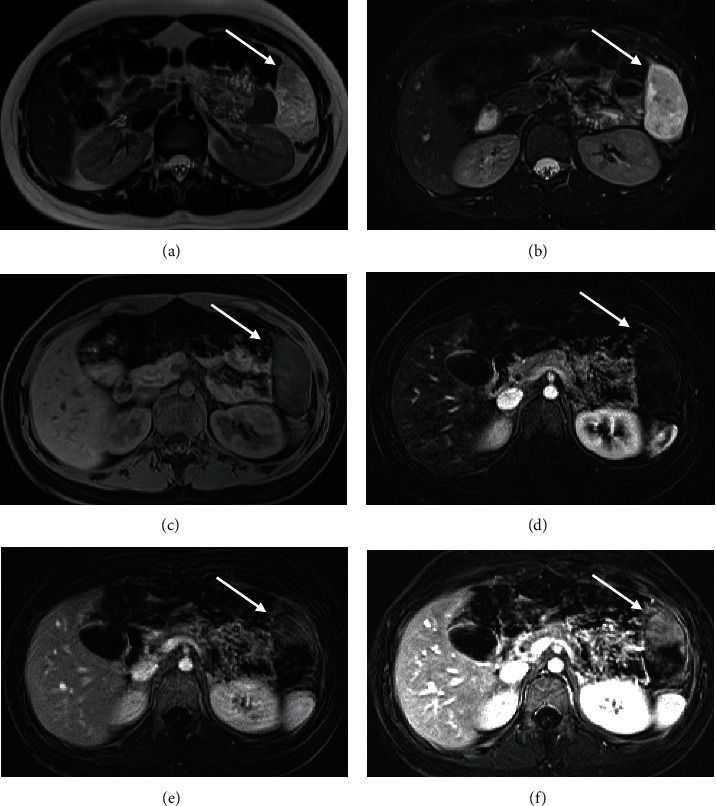
Axial T2-weighted image showing a left upper quadrant lesion with heterogenous hyperintense signal (arrow in (a)). Corresponding axial T2-weighted with fat saturation which shows no evidence of fat signal suppression within the lesion (arrow in (b)). Corresponding T1-weighted image with fat saturation showing no signal hyperintensity excluding the presence of methemoglobin (arrow in (c)). Dynamic contrast-enhanced sequences with subtraction technique (d–f) showed faint early arterial enhancement (arrow in (d)) which progressed in the venous phase (arrow in (e)) and continued to increase in signal intensity during equilibrium phase (arrow in (f)).

**Figure 3:**
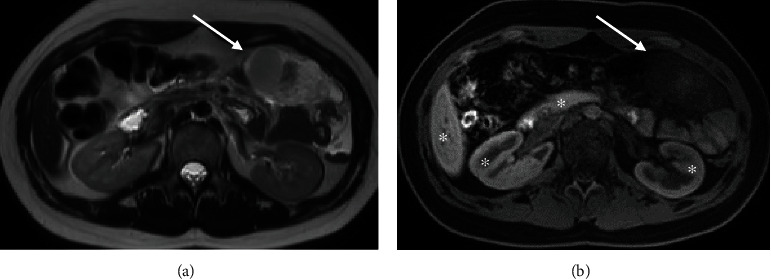
Follow-up noncontrast MRI 18 hours after the initial MRI study. Axial T2-weighted image shows an anterior shift in position of the lesion which remains heterogenous with predominantly hyperintense signal (arrow in (a)). There are no new areas of significant signal loss to suggest deoxyhemoglobin formation. Corresponding axial T1 fat sat image shows no retention or layering of contrast from the prior contrast-enhanced MRI exam (arrow in (b)). There remains no evidence of hyperintense signal to suggest methemoglobin formation. Note residual contrast enhancement of the liver, pancreas, and kidneys (asterisks in (b)).

The following day, the abdominal MRI redemonstrated an intraperitoneal mass in the left upper quadrant (Figure 2) without significant change in size. The mass, however, showed clear interval superior migration and rotation from the central left abdomen to the RUQ, suggesting a mesenteric origin with no adherence to the adjacent organs. The mass displayed slightly heterogenous low T1 and predominantly high T2 signal intensity. There was no suppression of the T2 signal hyperintensity with fat saturation application to suggest the presence of lipids. Dynamic gadolinium-enhanced images using the background subtraction technique showed subtle early and progressive enhancement of the nondependent and peripheral margins suggestive of a vascularized solid mass. Additional enhancing mesenteric lesions were also identified in the left upper quadrant, measuring up to 1.1 cm and 0.7 cm, which raised the possibility of satellite neoplastic implants. As an additional cautionary measure, given the patient's young age and recent history of trauma, a follow-up noncontrast MRI was obtained to assess for contrast extravasation and any evidence of MRI signal evolution that could support the diagnosis of traumatic hematoma with active bleeding. The delayed MRI obtained 18 hours after initial MR imaging and 32 hours after initial CT showed no signal evolution of the lesion on the T2-weighted images or evidence of contrast extravasation to suggest active bleeding (Figure 3).

Laparoscopic surgery was performed, which showed that the primary mass was between the greater omentum leaves that extend between the transverse colon and the stomach. Without evidence of mesocolon, stomach, or colon invasion, a successful resection of the mass was executed. An omental satellite lesion measuring 1.5 cm near the larger mass was excised, including two additional subcentimeter lesions confirming observations seen on MRI. Total blood loss was 10 ml, and the patient was discharged the day after surgery, indicating a rapid and uneventful postoperative recovery.

**Figure 4:**
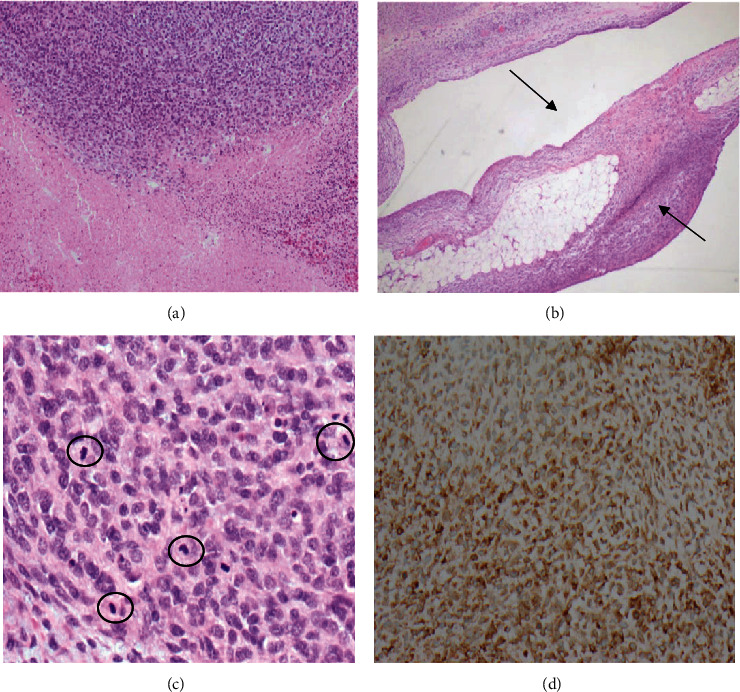
Histopathological images of the resected tumor. Areas of extensive tumor necrosis (H&E ×100) (a). Photomicrograph shows tumor located within omental adipose tissue (H&E ×100) (arrows in (b)). Cellular spindle tumor areas with frequent mitoses (highlighted in the circles) (H&E ×400) (c). PanTRK immunostain shows diffuse cytoplasmic staining (×400) (d).

A gross examination of the resected tumor (9 × 5.5 × 4 cm) revealed a nodular soft tissue mass with surrounding membranous tissue, focally haemorrhagic cut surfaces with areas of necrosis and possible calcifications. Microscopy revealed a lobulated cellular tumor with interlacing bundles of monotonous spindle to ovoid cells, loose myxoid areas, extensive necrosis, and focal dystrophic calcifications (Figure 4(a)), involving the lobules of omental fat (Figure 4(b)). Mitoses are frequent, 44 mitoses per 10 high power field (Figure 4(c)).

Immunohistochemistry analysis showed that the tumor cells were positive for beta-catenin (nuclear staining), inhibin, bcl2, and PanTRK, while PAX8 demonstrated a patchy positivity of some spindle cell populations. Most other type and site-specific markers were negative. Since the NTRK-rearranged tumors usually show positivity with PanTRK antibodies (Figure 4(d)), the molecular analysis using next-generation sequencing (NGS) was performed. Based on the histopathological results, the case was diagnosed as high-grade sarcoma with fibrosarcoma-like pattern. Subsequent NGS analysis detected a structural genomic rearrangement leading to the KMT2D : BCOR fusion (t(12; X)(12q13.11;Xp11.4)), and the pathological staging was determined as pT2pNxpMx.

FDG-PET/CT performed two months after surgery revealed 5 foci of tracer uptake in the left upper quadrant and pelvis consistent with hypermetabolic mesenteric tumor implants consistent with previously undetectable deposits and/or disease progression. There was no detectable metastatic disease. Clinical staging as per the AJCC 8^th^ edition classification system was stage III A (T2 N0 M0, G 2/3). Three weeks after the FDG-PET/CT, the patient received 3-days of ifosfamide and doxorubicin induction therapy, with concurrent mesna and dexrazoxane administration.

## 3. Discussion

A case report of a 14-year-old child with abdominal high-grade fibrosarcoma was presented in this study. Infantile fibrosarcoma is a rare malignancy that metastasizes infrequently among infants and young children and constitutes less than 1 percent of all childhood cancers. In contrast, the adult form is highly invasive and can affect older children, usually between 10 to 15 years old. Adult fibrosarcoma accounts for nearly 7% of all sarcoma diagnoses [5–8]. This type of tumor mainly affects the extremities, so intra-abdominal cases are sporadic.

In our case, the initial CT findings suggested that the detected mass was more likely to be a traumatic hematoma as the patient was involved in an ATV crash and experienced blunt abdominal trauma. Acute mesenteric hematomas can have well-defined margins and attenuation ranging from 40 to 70 HU [9], like our case. However, it is essential to note that it is not uncommon for patients with an incidentally noted soft tissue tumor such as fibrosarcoma to be misdiagnosed as a traumatic hematoma due to its association with recent trauma [10, 11]. To exclude a possible underlying neoplasm, dynamic contrast enhancement MRI with background subtraction technique is generally recommended to detect the presence of solid enhancement optimally. Due to the high sensitivity of this technique, the lack of contrast enhancement essentially excludes a mass lesion [12]. However, it should also be noted that slow progressive internal and peripheral enhancement is not specific for neoplasm, as it has been shown in expanding chronic hematoma with capillary ingrowth [12], adding to the chances of a false positive diagnosis of neoplasm in this case.

To further confound interpretation, differentiating hematomas from soft tissue tumors on noncontrast sequences can be challenging given the variable appearance and signal intensities in hematomas depending on the stage of metabolism [13–16]. In the hyperacute phase of hematoma (<24 h), there is typically oxyhemoglobin present in intact red blood cells, causing a hematoma to appear hypointense on T1-weighted and hyperintense on T2-weighted images [13, 15, 16], mimicking some neoplasms. Fortunately, in the acute hematoma phase (1-3 days), rapid progression to deoxyhemoglobin leads to substantial loss of T2 signal intensity, highlighting the importance of serial MRI in this case. The follow-up MRI study occurring greater than 32 hours following the traumatic injury failed to show any expected T2 signal loss to suggest the formation of deoxyhemoglobin. Further metabolism to methemoglobin is characterized by T1-weighted hyperintensity characteristic of the early subacute phase (3 to 7 days) of hematoma [13, 15], which was also not present in our case. The overall lack of volumetric expansion, contrast extravasation, or signal intensity change on repeat MRI helped to exclude an actively bleeding hyperacute hematoma confidently.

In histologically examining the tumors with fibrosarcoma-like patterns, malignant fibroblasts are the predominant cell type with variable collagen production levels. Given the morphologic heterogeneity of these tumor types, diagnosis is often based on immunohistochemical and molecular diagnostic markers [17]. In our case, the tumor pathology demonstrated the typical features of high-grade fibrosarcoma, including interlacing fascicles of spindled to ovoid tumor cells with loose myxoid areas and lack of immunoreactivity with myogenic markers. Infantile fibrosarcoma is usually driven by gene fusions involving the NTRK (neurotrophic receptor tyrosine kinase) gene family. Based on the World Health Organization (WHO) 2013 definition, those tumors that are histologically diagnosed as infantile fibrosarcoma but lacking a characteristic NTRK fusion could be considered infantile fibrosarcoma-like tumor [18]. PanTRK immunohistochemistry is a prescreening technique for detecting tumors with a high prevalence of *NTRK* fusions, such as infantile fibrosarcoma [19]. However, confirmation with a molecular method such as NGS is necessary. Our NGS analysis revealed a rare chromosomal rearrangement resulting in KMT2D : BCOR fusion. Tumors harboring rare fusion variants of BCOR have been described before. For example, Kao et al. have reported tumors with KMT2D : BCOR genotype showing a fusion transcript of exon 39 of KMT2D (12q13.12) to exon 6 of BCOR (Xp11.4) as well as positivity for PanTRK staining and NTRK3 overexpression [20].

The role of FDG-PET/CT in diagnosis of soft tissue sarcomas is not well established as there remain challenges in differentiating malignant from benign lesions [21]. However, due to the sensitivity to tumors with high metabolic activity, such as high-grade sarcomas, as found in our case, FDG-PET/CT improves the likelihood of detecting small regional deposits or distant metastatic disease in the initial workup period and plays an important role in identifying or excluding local relapse or metastasis essential in the long-term management of these patients [22, 23].

In conclusion, we report a rare case of a primary high-grade mesenteric sarcoma incidentally identified on initial CT evaluation following traumatic injury to the abdomen. Due to the potential overlapping appearance between neoplasms and hematomas, this case illustrates the importance of dynamic contrast-enhanced serial MRI as a problem-solving tool when the less intuitive diagnosis of neoplasia is considered in the post-traumatic setting.

## Data Availability

No underlying data was collected or produced in this study.
